# Active Nuclear Receptors Exhibit Highly Correlated AF-2 Domain Motions

**DOI:** 10.1371/journal.pcbi.1000111

**Published:** 2008-07-11

**Authors:** Denise G. Teotico, Monica L. Frazier, Feng Ding, Nikolay V. Dokholyan, Brenda R. S. Temple, Matthew R. Redinbo

**Affiliations:** 1 Department of Chemistry, University of North Carolina at Chapel Hill, Chapel Hill, North Carolina, United States of America; 2 Department of Biochemistry and Biophysics, University of North Carolina at Chapel Hill, Chapel Hill, North Carolina, United States of America; 3 The Lineberger Comprehensive Cancer Center, University of North Carolina at Chapel Hill, Chapel Hill, North Carolina, United States of America; 4 University of North Carolina RL Juliano Structural Bioinformatics Core Facility, Chapel Hill, North Carolina, United States of America; Wake Forest University, United States of America

## Abstract

Nuclear receptor ligand binding domains (LBDs) convert ligand binding events into changes in gene expression by recruiting transcriptional coregulators to a conserved activation function-2 (AF-2) surface. While most nuclear receptor LBDs form homo- or heterodimers, the human nuclear receptor pregnane X receptor (PXR) forms a unique and essential homodimer and is proposed to assemble into a functional heterotetramer with the retinoid X receptor (RXR). How the homodimer interface, which is located 30 Å from the AF-2, would affect function at this critical surface has remained unclear. By using 20- to 30-ns molecular dynamics simulations on PXR in various oligomerization states, we observed a remarkably high degree of correlated motion in the PXR–RXR heterotetramer, most notably in the four helices that create the AF-2 domain. The function of such correlation may be to create “active-capable” receptor complexes that are ready to bind to transcriptional coactivators. Indeed, we found in additional simulations that active-capable receptor complexes involving other orphan or steroid nuclear receptors also exhibit highly correlated AF-2 domain motions. We further propose a mechanism for the transmission of long-range motions through the nuclear receptor LBD to the AF-2 surface. Taken together, our findings indicate that long-range motions within the LBD scaffold are critical to nuclear receptor function by promoting a mobile AF-2 state ready to bind coactivators.

## Author Summary

Long-range motions play essential roles in protein function but are difficult to appreciate from static crystal structures. We sought to understand how macromolecular motion affects the formation of transcriptional complexes central to controlling gene expression. Using 20- to 30-ns molecular dynamics simulations, we examined three nuclear receptors that function as ligand-regulated transcription factors: the pregnane X receptor, the peroxisome proliferator-activator receptor-γ, and estrogen receptor-α. We found that each of these receptors exhibits a high degree of correlated motions within the domain responsible for forming functionally essential protein–protein interactions with transcriptional coactivators. We further found that specific long-range (up to 30 Å) motions play an important role in these dynamics. Our results show that “active-capable” nuclear receptors are prepared for coactivator contacts by maintaining a mobile but preformed protein–protein interaction surface.

## Introduction

The nuclear receptor (NR) superfamily of ligand-regulated transcription factors controls the expression of genes essential to metabolism, development and systemic homeostasis [1]–[3]. NRs are modular proteins typically composed of a conserved N- terminal Zn-module DNA binding domain (DBD) that targets specific response elements, a variable hinge region, and a C-terminal ligand binding domain (LBD) capable in most cases of responding to specific small molecule ligands [4]. NR LBDs contain a shallow activation function 2 (AF-2) surface formed by helices α3, α3′, α4 and αAF that is essential for ligand-dependent interactions with transcriptional coregulators. The AF-2 surface complexes with LxxLL-containing transcriptional coactivators in the presence of agonist ligands, and with distinct leucine-rich corepressor motifs in the presence of antagonists or in the absence of ligand [4],[5].

**Figure 1:**
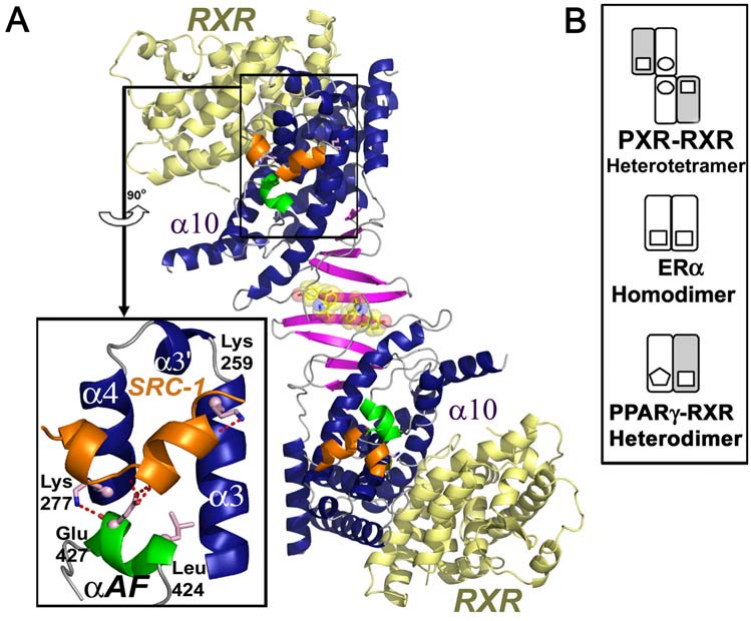
Structural Features of the PXR-RXR Heterotetramer. (A) A model of the PXR (blue, magenta, green)-RXR (yellow) heterotetramer highlights the PXR homodimer interface and the ten-stranded intermolecule β-sheet formed between the two monomers. PXR residues Trp-223 and Tyr-225 central to homodimerization are rendered in yellow with transparent CPK spheres. The α-helices 3, 3′, 4 and αAF (green) create the AF-2 surfaces that bind leucine-rich coactivator peptides like SRC-1 (orange) using a charge clamp (Lys-259, Glu-427) and other residues (light pink). (B) Schematics of the oligomeric NR complexes examined in this paper.

The pregnane X receptor (PXR) controls the expression of a wide range of gene products involved in xenobiotic metabolism and endobiotic homeostasis [6]–[8], and is unusual in the NR superfamily in several respects. First, PXR responds promiscuously to a wide range of chemically-distinct ligands from small lipophilic phenobarbital (232 Da) to the large macrolide antibiotic rifampicin (823 Da); in contrast, most NRs are highly specific for their cognate ligands [9]–[11]. Second, the PXRs of known sequence contain a 50–60 residue insert that, as observed in human [12]–[14], creates a unique β-turn-β motif and novel PXR homodimer interface. All NR LBDs fold into a three-layer α-helical sandwich in which α10 forms standard homodimerization interactions (*e.g.*, for steroid receptors like the estrogen receptor-α, ERα) or heterodimerization interactions (*e.g.*, with RXR for orphan receptors like PXR) [2],[15],[16]. The PXR LBD, in contrast, contains a second oligomerization interface at the novel β-turn-β motif in which intercalating tryptophan and tyrosine residues (Trp-223/Tyr-225) lock across the dimer to form an aromatic zipper [4],[5],[12] (Figure 1A). It has been shown that this dimer interface is essential to PXR function, and that the specific disruption of homodimerization eliminates the ability of the receptor to interact with transcriptional coactivators like steroid receptor coactivator 1 (SRC-1), but does not impact PXR's subcellular localization or its association with DNA, RXR, or activating ligands [12]. This work led to the proposal of a PXR-RXR heterotetramer as the functional unit [12] (Figures 1A, 1B).

The unique PXR homodimer interface, however, is located more than 30 Å from the coactivator binding site at the receptor's AF-2 surface (Figure 1A). Thus, we hypothesize that long-range motions within the PXR LBD are essential for communicating the stabilizing effect of PXR homodimerization to the AF-2 domain. To test this hypothesis, we performed all-atom molecular dynamics (MD) simulations on both the PXR LBD, as well as two other nuclear receptor LBDs, in various states (Table 1). The former orphan peroxisome proliferator-activated receptor-γ (PPARγ) is functional as a heterodimer with RXR, while the steroid estrogen receptor-α (ERα ) is active as an analogous homodimer (Figure 1B). We examined LBDs in inactive states (*e.g.,* monomers or mutants), as well as those in the proper functional states (*e.g.,* homo- or heterodimers, or as a heterotetramer for RXR) we have termed “active-capable.” Our results support the conclusion that the NR LBD provides a scaffold for long-range motions that prepare the AF-2 surface for binding to transcriptional coactivators.

**Table 1:** Summary of MD Simulations.

Receptor/Oligomeric State	PDB ID	Length of Simulation		Activity of State
		Total Time	Period Used in Analysis	
PXR-RXR heterodimer*	1ILG	30 ns	20–30 ns	Inactive
PXR-RXR heterotetramer*	1ILG	30 ns	20–30 ns	Active-capable
PPARγ467L-RXR heterodimer	1RDT**	20 ns	10–20 ns	Inactive
PPARγ-RXR heterodimer	1RDT	20 ns	10–20 ns	Active-capable
ERα monomer	1ERE	20 ns	10–20 ns	Inactive
ERα dimer	1ERE	25 ns	15–25 ns	Active-capable

*: All PXR simulations are based on 1ILG with residues 178–197 modeled in InsightII.

**: Single-site mutant of PPARγ generated in Pymol. There is no crystal structure of the mutant.

## Results

### Stable Dynamic Trajectories

**Figure 2:**
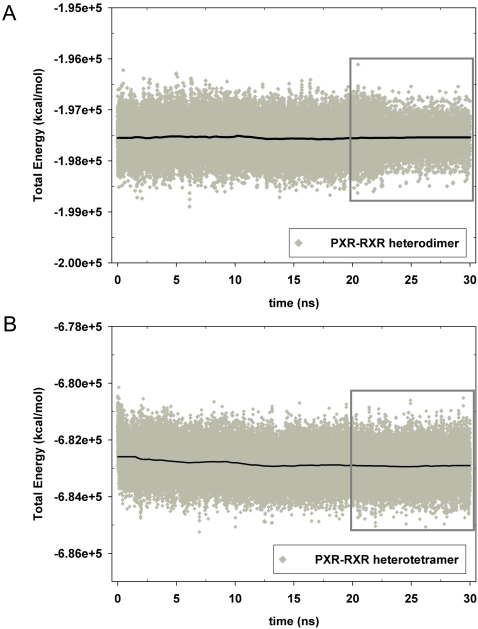
Conservation of Total Energy During PXR-RXR Simulations. Total energy (kcal/mol), used as a measure of overall simulation stability, remains relatively constant during the course of both the PXR-RXR heterodimer (A) and PXR-RXR heterotetramer (B) simulations, particularly during the final 10 ns used for analysis (boxed). Both the total energy (grey diamonds) and a running average (black line) are shown.

Six all-atom molecular dynamics (MD) runs were performed for 20–30 ns on three nuclear receptor LBDs (Table 1). PXR was examined both as a heterodimer with RXR and as a heterotetramer with RXR (30 ns simulations). Wild-type PPARγ was examined as a heterodimer with RXR, and the inactive PPARγ P467L mutant was also examined as a heterodimer with RXR (20 ns simulations). Finally, ERα was examined both in its inactive monomeric state (20 ns), and as an ERα homodimer (25 ns). All six trajectories were judged as stable by two criteria. First, the total energy of each system, calculated as the sum of kinetic and potential energy at each time point, was found to be essentially constant after the first 2–3 ns (Figure 2, Figure S1). These results indicate that after a short period of equilibration, each simulation was sampling an energetically stable conformational ensemble. Second, all trajectories were analyzed in terms of moving average all-atom root mean square deviations (RMSDs) from starting crystal structures over the simulation time course (Figures S2, S3). The PXR-RXR trajectories exhibited RMSD values of 0.7–5.0 Å (Figure S2), while the PPARγ- and ERα-containing trajectories exhibited values of 1.7–3.5 Å (Figure S3). Such deviations were considered low for systems of this size (*e.g.,* 1044 residues for the PXR-RXR heterotetramer). The RMSD results indicate that all simulations were stable for at least the last 10 ns of each trajectory (Figures S2, S3). Thus, the final 10 ns section of each simulation was used for subsequent analysis.

### Highly Correlated Motion in the PXR-RXR Heterotetramer

**Figure 3:**
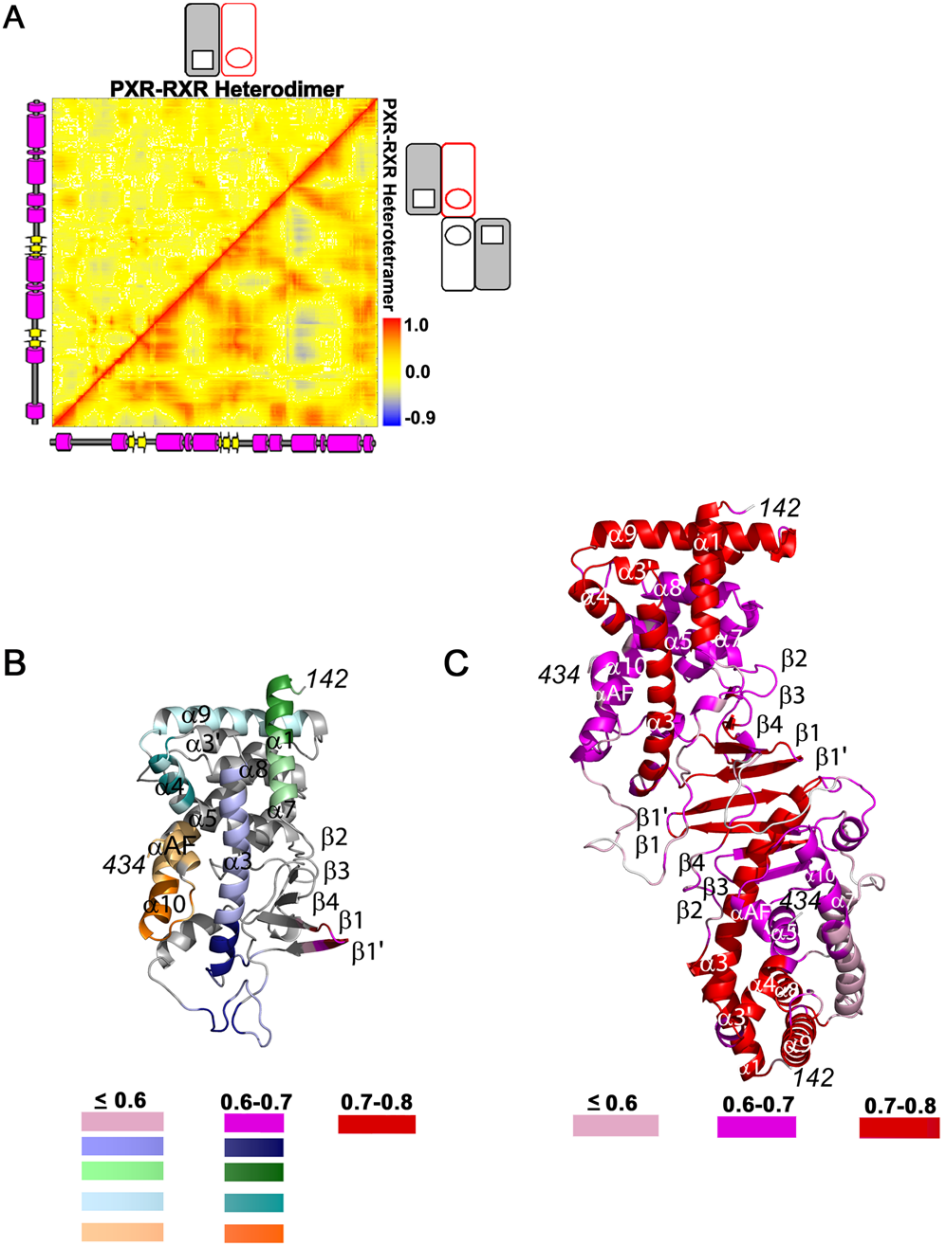
Highly Correlated Motion in the PXR-RXR Heterotetramer. (A) Covariance analysis of the PXR LBD in the PXR-RXR heterodimer and heterotetramer. Residue-residue correlation coefficient values range from blue (anticorrelated, –0.9) to red (correlated, +1), with uncorrelated residue pairs in yellow. Secondary structure is provided from right-to-left, and bottom-to-top. (B) Clustering of correlated PXR LBD residues from the PXR-RXR heterodimer simulation. Eleven clusters were identified, five with a correlation cutoff (CC) of 0.6, five with a CC of 0.7, and one with a CC of 0.8. (C) Clustering of correlated PXR LBD residues from the PXR-RXR heterotetramer simulation. Three clusters were identified, one each with CCs of 0.6, 0.7, and 0.8. Clusters are colored by the maximum correlation coefficient at which they are observed.

The PXR-RXR heterotetramer is expected to have distinct functional dynamics relative to the heterodimer because the heterotetramer contains the unique PXR homodimer interface shown to be essential for receptor activity [12] (Figure 1). Thus, we examined the PXR LBDs in both the PXR-RXR heterodimer and PXR-RXR heterotetramer simulations over the last 10 ns of each trajectory using essential dynamics analysis. Essential dynamics discriminates between concerted motions of residue clusters within a protein and uncorrelated residue fluctuations [17]. We computed normalized covariance matrices [18] to classify the relationships between all possible residue pairs in the protein (Figure 3A). In this analysis, correlation (two residues moving in the same direction) is indicated by residue-residue correlation coefficients approaching +1, while correlation coefficients approaching −1 indicate anticorrelation (residues moving in opposite directions). Correlation coefficients near zero, in contrast, are associated with residue pairs that lack a dynamic relationship.

The PXR LBDs in the PXR-RXR heterotetramer exhibit significantly more residue-residue correlation relative to the PXR LBD in the PXR-RXR heterodimer (Figure 3A). Indeed, the distribution of correlation coefficients for the PXR LBD in the PXR-RXR heterodimer has one peak centered close to zero, indicating the majority of residue-pairs are not correlated (data not shown). In contrast, the correlation coefficient distribution for the PXR LBDs in the PXR-RXR heterotetramer has two distinct peaks, one positive and one negative, indicating both residue-residue correlation and anticorrelation (data not shown).

Clusters of correlated PXR residues from the PXR-RXR heterodimer and heterotetramer that exhibited concerted motion were then examined for the strength of their residue-residue correlation coefficients and the biological significance of those dynamics. Clustering the PXR LBDs from the PXR-RXR heterotetramer at a correlation coefficient less than 0.6 produced a single cluster containing the complete PXR-RXR homodimer, while clustering at a correlation coefficient above 0.8 resulted in clusters comprised of only 2–5 residues. Neither coefficient cutoff alone could interrogate the biological relevance of the concerted motions; thus, we classified clusters using three correlation coefficient cutoffs (Figure 3B, C). Such cutoffs discriminate between weak (0.6 or less), medium (between 0.6 and 0.7), and strong (between 0.7 and 0.8) correlations between PXR residues. The same cutoffs set in the heterotetramer were used, for consistency, to cluster the relatively weak correlated motion observed in the PXR-RXR heterodimer. Indeed, the PXR LBD from the heterodimer exhibited only five small correlated clusters, with smaller regions of these weakly correlated clusters remaining at a medium strength correlation coefficient, and only one group of a few residues identifiable at a strong correlation coefficient (Figure 3B).

In distinct contrast, however, the PXR LBDs in the PXR-RXR heterotetramer form a strongly correlated unit (Figure 3C). The β-sheet region involved in the PXR-PXR homodimerization interface (β1, β1′, β3, β4), together with α-helices 1, 3, 3′, 4 and 9, exhibit the strongest degree of correlation; the residues of these β-sheets and α-helices are all clustered together with a correlation coefficient of 0.8. The neighboring helices, including αAF, also exhibit highly correlated motion with correlation coefficients >0.6 (Figure 3C). The strength of residue-residue correlations throughout this region suggest that α3 forms a critical conduit through which the stabilizing effects of the homodimer interface involving β1, β1′, β3, and β4 are communicated to helices 3, 3′, 4 and AF of the AF-2 surface. In the PXR-RXR heterodimer, however, the same β-sheet region is anticorrelated with the AF-2 domain (Figure 3B).

### Highly Correlated Motion in the PXR-RXR Heterotetramer AF-2 Surface

We next examined the motions in the four helices that create the AF-2 coactivator binding surface on PXR: α3, α3′, α4, and αAF. The concerted motion of this surface was compared between the PXR LBDs in the PXR-RXR heterodimer and heterotetramer trajectories, and was examined using both quasiharmonic analysis (QHA) and normal mode analysis (NMA). Both methods have benefits and limitations. For quasiharmonic analysis, its benefits are all-atom resolution and the use of explicit solvent, but it is limited by the time constraints of all-atom MD. Normal mode analysis has the benefit of observing motions on a longer timescale than available with QHA, but is limited to analyses based upon the coarse grained model solely of the macromolecule. Our results agree with others that it takes more NMA modes than QHA modes to describe the same motions [19]. Thus, we employed the first two modes from QHA and first 14 nontrivial modes from NMA (see Methods). Eigenvectors from these analyses are associated with the magnitude and direction of motion, and these eigenvectors can be used to create visuals of the NR's motion.

**Figure 4:**
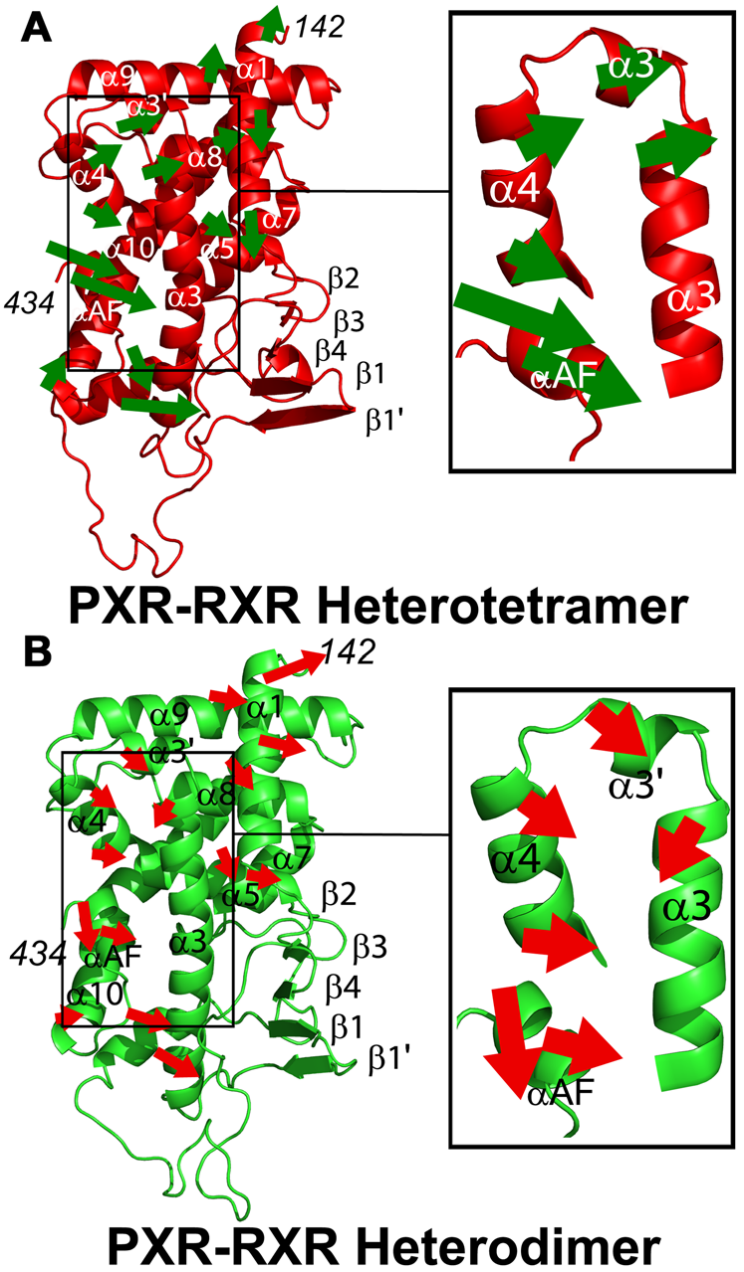
Correlated AF-2 Domain Motions in the PXR-RXR Heterotetramer. Vectors describing the motions of PXR LBD α-helices from the heterotetramer (A) and heterodimer (B) simulations show the active-capable heterotetramer PXR LBD exhibits more overall correlated motion as well as correlation between AF-2 surface helices. Each helix eigenvector (shown by an arrow) is the sum of the α-carbon eigenvectors in that helix. All arrows were generated using the same scalar magnifications of motion vectors and are presented on the same scale. As such, they represent relative, rather than absolute, movements.

After examining the vectors describing the primary modes of motion derived from QHA for each α-carbon position in the PXR LBDs of the PXR-RXR simulations, a single average vector was calculated to describe the motion of seven of the eleven α-helices in the LBD. The remaining four helices, α3, α4, αAF and α10, displayed distinct motions at their termini; thus, for these helices, two average vectors were employed. The results of this analysis show that the PXR LBD helices from the PXR-RXR heterotetramer move as a single unit, and in one direction (Figure 4A). This correlation is especially evident in the AF-2 surface, as α3, α3′, α4 and αAF all move together in the same direction (Figure 4A inset). In contrast, the PXR LBD from the PXR-RXR heterodimer exhibited relatively small, disjointed motions (Figure 4B). This lack of helix-helix correlation includes the AF-2 surface helices α3, α3′, α4 and αAF (Figure 4B inset).

AF-2 mobility identified by QHA was also assessed by examining the angles between the directions of motion as defined by the eigenvectors for α-carbons of residues important to coactivator binding (Table 2, Methods). As such, if two residues in the AF-2 surface are moving together, the angle between them is small (see Methods, Equation 1). The average angle from the sum of motion vectors (modes) 1 and 2 between AF-2 domain residues in the PXR-RXR heterodimer simulation was 71.6°. In contrast, the average angle for the same residue pairs in the PXR-RXR heterotetramer simulation was 31.5° (Table 2). Taken together, these QHA results support the conclusion that the intramolecular β-sheet formed by the PXR homodimer interface produces highly correlated AF-2 surface motions in the PXR-RXR heterotetramer complex.

**Table 2:** θ Angle Analysis of α-carbons of PXR LBD.

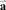	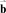	QHA		NMA	
		Active-capable	Inactive	Active-capable	Inactive
		PXR from Heterotetramer	PXR from Heterodimer	PXR from Heterotetramer	PXR from Heterodimer
**Lys277 (**α**4)**	**Lys259 (**α**3)**	21.0°	70.2°	7.4°	84.5°
**Lys259 (**α**3)**	**Glu427 (**α**AF)**	47.9°	83.6°	9.7°	104.7°
**Lys259 (**α**3)**	**Leu424 (**α**AF)**	43.6°	65.1°	23.4°	133.3°
**Lys277 (**α**4)**	**Glu427 (**α**AF)**	31.5°	66.9°	6.4°	20.6°
**Lys277 (**α**4)**	**Leu424 (**α**AF)**	32.1°	99.0°	20.2°	48.8°
**Leu424 (**α**AF)**	**Glu427 (**α**AF)**	12.8°	45.0°	13.9°	28.8°
**Average**		**31.5**°	**71.6**°	**13.5**°	**70.1**°

In a second analysis, modes of motion of the AF-2 surface of the PXR LBD were examined from both the heterodimer and heterotetramer trajectories using NMA. Similar to the QHA study above, angles between the directions of motion as defined by the eigenvectors for α-carbons of residues important to coactivator binding were calculated (Table 2, Methods). The average angle observed in the AF-2 surface in the PXR-RXR heterotetramer was 13.5° using NMA, even smaller than the average angle found using QHA (Table 2). In contrast, the average angle for the same PXR AF-2 residues in the PXR-RXR heterodimer was 70.1°, nearly identical to the value found using QHA (Table 2). Thus, these data support the conclusions of the QHA study, and indicate that a high degree of helix-helix correlation is present in the AF-2 surface of the PXR-RXR heterotetramer relative to the heterodimer. Similarities between the QHA and NMA results strengthen this collective conclusion, particularly because QHA is based on shorter dynamic movements of all atoms, while NMA examines harmonic oscillations that occur on longer time scales.

**Figure 5:**
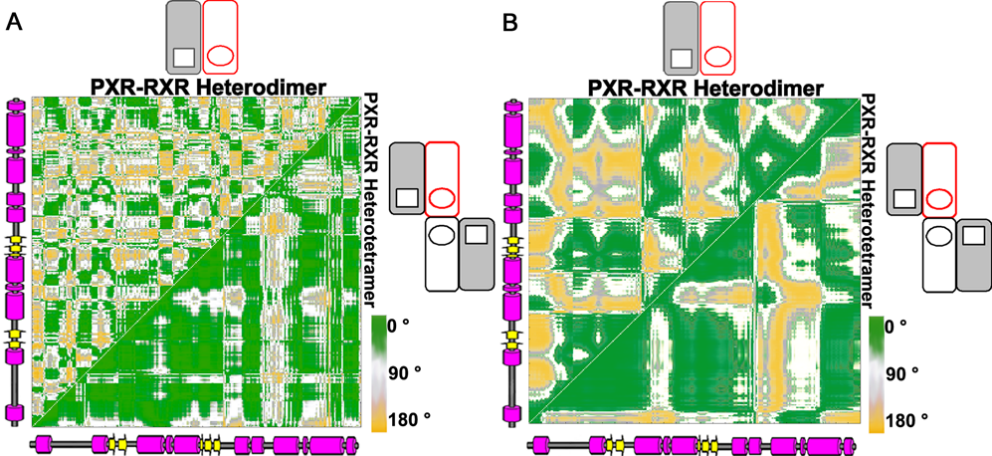
Quasiharmonic and Normal Mode Analyses. Angles between motion vectors for all residue pairs in the PXR-RXR heterodimer and heterotetramer. Motion vectors were identified by quasiharmonic analysis (QHA, using the first two modes; (A) and by normal mode analysis (NMA, using the first 14 nontrivial modes; (B). In the plots, green represents angles close to zero (correlated), while yellow indicates angles close to 180° (anticorrelated).

Plots of the angles between the vectors of motion of all possible PXR LBD residue pairs from both the heterodimer and heterotetramer simulations for the QHA and NMA studies are shown in Figures 5A and 5B, respectively. Areas in green represent angle values close to zero (vectors moving in the same direction, or correlated), while areas in yellow indicate vectors with angles close to 180° (vectors moving in the opposite direction, or anticorrelated). In both plots, a high degree of correlated motion is observed for the PXR LBD in the PXR-RXR heterotetramer, while significantly less correlation is observed for the LBD in the heterodimer (Figures 5A, B). The similarity between Figures 5A and B, from selected modes of QHA- and NMA-identified motion, and Figure 3A, from all modes of motion, indicates that enough modes were chosen in both QHA and NMA to represent the motion of each LBD (Methods). In addition, both the QHA and NMA plots for the heterotetramer indicate similar correlated structural elements. For example, the PXR β-sheet moves in a more correlated manner with respect to αAF in the heterotetramer relative to the heterodimer (Figures 5A, B). In summary, long-range motions impacted by the oligomeric state of PXR play a central role in the function of this nuclear xenobiotic receptor.

### Correlated AF-2 Motions in Other Nuclear Receptors

We next examined whether the unliganded LBDs of other members of the NR superfamily would also exhibit correlated AF-2 surface motions. As stated above, 20–25 ns MD simulations were performed on two inactive NR states, the ERα monomer and the PPARγ P467L-RXR heterodimer complex, and on two “active-capable” states, the ERα homodimer and the wild-type PPARγ-RXR heterodimer. A P467L mutation has been shown to inactivate PPARγ [20]. Only moderate levels of residue-residue correlation and anticorrelation were observed for both states of ERα and PPARγ (Figures S4A, B). Examination of correlation coefficient distributions in these simulations reveals that all remain close to zero, indicating relatively non-correlated motion (data not shown).

**Figure 6:**
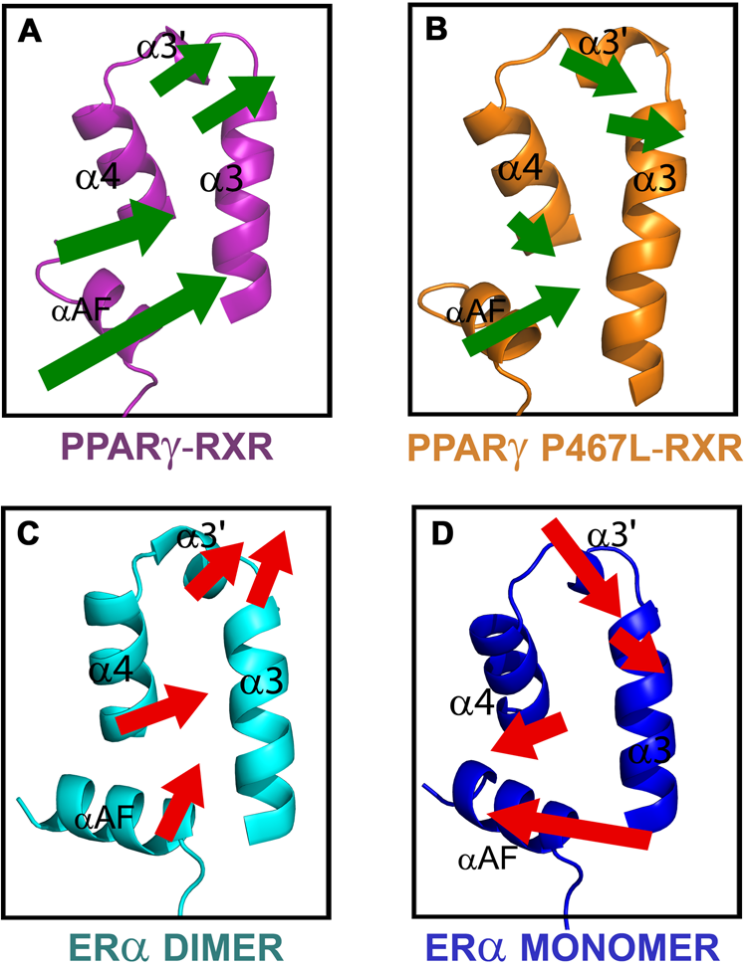
AF-2 Surface Motions in PPARγ and ERα Complexes. Similar to Figure 4, the active-capable PPARγ-RXR heterodimer and ERα homodimer complexes exhibit correlated motions in their AF-2 surfaces during MD trajectories (A, C), while inactive states of both receptors exhibit reduced AF-2 surface correlation (B, D).

In spite of their relatively limited overall correlation, however, the active-capable forms of ERα and PPARγ-RXR exhibited correlated AF-2 domain motions. Similar to the analysis of the PXR trajectories, both QHA and NMA were employed to examine these ERα and PPARγ simulations. Results from QHA studies reveal that the active-capable forms of ERα, and PPARγ exhibit more correlated AF-2 motions than their inactive counterparts (Figure 6). Angles between the vectors describing AF-2 surface helix motions in PPARγ and ERα states using both QHA and NMA further support the overall conclusion that active-capable states exhibit correlated AF-2 surfaces (Table 3, 4). For example, the average angles for ERα homodimer and wild type PPARγ-RXR determined using NMA are 41.0° and 48.8°, respectively, while those for the inactive ERα monomer and the PPARγ P467L mutant are 63.1° and 58.3°. Again, the AF-2 correlation in motion observed using the shorter time scales of all-atom molecular dynamics simulations and QHA are also seen in the longer harmonic oscillations of NMA. In summary, correlated motion appears to be a consistent feature in the AF-2 domains of active-capable nuclear receptor LBDs.

**Table 3 pcbi-1000111-t003:** θ Angle Analysis of α-carbons of PPARγ LBD.

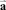	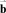	QHA		NMA	
		Active-capable	Inactive	Active-capable	Inactive
		WT PPARγ-RXR	P467L PPARγ-RXR	WT PPARγ-RXR	P467L PPARγ-RXR
**Lys277 (**α**4)**	**Lys259 (**α**3)**	3.9°	39.4°	29.2°	0.9°
**Lys259 (**α**3)**	**Glu427 (**α**AF)**	1.8°	40.2°	61.8°	35.8°
**Lys259 (**α**3)**	**Leu424 (**α**AF)**	1.2°	37.0°	79.6°	104.1°
**Lys277 (**α**4)**	**Glu427 (**α**AF)**	4.0°	70.1°	44.2°	36.0°
**Lys277 (**α**4)**	**Leu424 (**α**AF)**	3.0°	49.2°	60.3°	104.1°
**Leu424 (**α**AF)**	**Glu427 (**α**AF)**	1.3°	26.6°	17.9°	68.7°
**Average**		**2.5**°	**48.8**°	**48.8**°	**58.3**°

**Table 4:** θ Angle Analysis of α-carbons of ERα LBD.

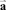	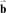	QHA		NMA	
		Active-capable	Inactive	Active-capable	Inactive
		ERα Dimer	ERα Monomer	ERα Dimer	ERα Monomer
**Lys277 (**α**4)**	**Lys259 (**α**3)**	26.0°	117.8°	47.0°	48.9°
**Lys259 (**α**3)**	**Glu427 (**α**AF)**	52.1°	94.5°	59.8°	83.4°
**Lys259 (**α**3)**	**Leu424 (**α**AF)**	47.0°	122.1°	58.1°	96.8°
**Lys277 (**α**4)**	**Glu427 (**α**AF)**	28.9°	54.1°	30.7°	59.2°
**Lys277 (**α**4)**	**Leu424 (**α**AF)**	64.0°	39.1°	38.0°	74.4°
**Leu424 (**α**AF)**	**Glu427 (**α**AF)**	51.4°	28.2°	12.6°	15.6°
**Average**		**44.9**°	**76.0**°	**41.0**°	**63.1**°

## Discussion

The differences in human PXR LBD motion between two oligomeric states of the receptor (as a heterodimer and a heterotetramer with RXR) were examined using molecular dynamics trajectories, essential dynamics, quasiharmonic, and normal mode analyses. It was hypothesized that the PXR heterotetramer, in which PXR LBD monomers form a unique homodimer shown to be critical for transcriptional regulation [12], would exhibit functionally-relevant motion. Indeed, we find that this “active-capable” form of PXR exhibits not only significantly more overall motion and more correlated motion relative to the heterodimer, but also highly correlated motion in the AF-2 surface responsible for functionally-essential contacts with transcriptional coactivators (Figures 4, 5). These data suggest that a high degree of motion promotes the proper function of this nuclear receptor, provided that the motion is correlated to preserve the state of the receptor ready to bind to leucine-rich coactivator motifs.

In addition, these results indicate that long-range motions are critical to the function of the xenobiotic receptor PXR. The homodimer interface unique to the PXR LBD is located approximately 30–35 Å from the AF-2 surface (Figure 1). Essential dynamics have revealed that the β-sheet and six α-helices in PXR (1, 3, 3′, 4, 9, AF), including those that comprise the AF-2 surface, move as a single unit in the heterotetramer trajectory (Figure 3). This suggests a structural mechanism by which PXR homodimerization creates a ten-stranded intermolecular β-sheet (Figure 1) that positively impacts AF-2 domain motion. The N-terminal portion of α3 appears to serve as a critical bridge between the PXR β-sheet and the AF-2 helices, such that correlated β1-β4 motion is “communicated” to α3-α4 and αAF (Figure 4). This relationship explains how the obligate PXR monomer mutant Trp-223-Ala/Tyr-225-Ala, in which the interlocking aromatic residues at the homodimer interface are eliminated, is still able to bind to ligand, DNA and RXR, but not to transcriptional coactivators at the AF-2 surface [12].

This hypothesized path of “communication through motion” mediated by α3 and involving several β-strands, as well as α1 and α9, correlates well with existing PXR structure-function data. First, Met-243, located in the N-terminal portion of α3, is contacted by ligands in all reported PXR LBD crystal structures [4],[21],[22]. Thus, they appear critical for the ligand-enhanced transcriptional activity exhibited by PXR. Second, single mutations in either α3 or α3′, such as Thr-248-Glu, Lys-277-Gln and Pro-268-His, result in a loss of PXR activity [23],[24]. In addition, although the α3 double-mutant Lys-277-Gln/Thr-248-Glu restores transcriptional activation, it abolishes the antagonism of ketoconazole, hypothesized to function by binding the AF-2 surface [24],[25]. Third, the α1 and α9 mutants Asp-163-Gly and Ala-370-Thr, respectively, represent a class of PXR variants that are distantly located from the AF-2 domain but result in reduced transcriptional activity [26]. Taken together, these data support the conclusion that the wild-type PXR LBD is “tuned” in its heterotetrameric complex with the RXR LBD to produce correlated motions that promote the binding of transcriptional coactivators.

Extension of this analysis into other nuclear receptors revealed correlated AF-2 surface motions in “active-capable” forms of ERα and PPARγ (Figure 6 and Figure S4). Thus, long-range motions may play critical roles in the LBD activation potential of several members of the nuclear receptor superfamily. Our results expand on previous MD investigations of NR LBDs. For example, dynamics studies on ERα [27] showed that the addition of coactivator peptide and ligand to apo ERα lead to increased αAF helix motion in unspecified directions. Similarly, studies on androgen insensitivity syndrome associated androgen receptor Pro-892-Ala and Pro-892-Leu mutations revealed via biochemical assays and MD simulations an increased flexibility and distortion of the αAF helix [28]. We present evidence that the AF-2 domain helices of the Erα, PPARγ, and PXR LBDs move together and in the same direction in each receptor. One may postulate that the uncorrelated motion between the helices in the AF-2 domain observed for inactive receptors (e.g., apo PXR-RXR heterodimer, ERα monomer and the PPARγ P467L-RXR mutant) may represent the initial transition towards an αAF position required for corepressor binding [29]. Alternatively, these anticorrelated motions may simply prevent coactivator binding to LBDs that are not in active-capable oligomeric states.

The results presented here are also in agreement with limited proteolysis [15], fluorescence polarization [20], and NMR [30],[31] studies that examined the stabilization of global and local motions of ERα [15] and PPARγ [20] upon ligand binding. Of particular note are time-resolved fluorescence polarization studies by Kallenberger and Schwabe [20] on the human P467L PPARγ mutant that causes insulin resistance and early onset hypertension. This mutation was found to weaken immobilization of αAF against the main body of the receptor. In our molecular dynamics simulations, wild type PPARγ-RXR exhibited a strong degree of correlated AF-2 motion while the PPARγ P467L-RXR mutant showed uncorrelated motion in its AF-2 domain (Figure 6C, D). This is the first model of nuclear receptor dynamics that relates changes in motion to a mutation causing a disease state.

While nuclear receptors are well-established targets for small molecule modulators that treat a wide range of conditions, current drugs function as agonists and antagonists via the ligand binding pocket. However, recent data have indicated that nuclear receptor LBDs can be antagonized using small molecules that block coregulator binding to the AF-2 surface. For example, thyroid receptor antagonists discovered by high-throughput screening were found to act at the AF-2 site of that receptor [32],[33]. In addition, the azole family of antifungal compounds has recently been shown to antagonize the action of human PXR via the AF-2 domain [24],[25]. The dynamics data presented here further elucidate the nature of motions essential for AF-2 active-capable function, and may facilitate the improved design or development of therapeutics targeted to specific NR AF-2 surfaces.

## Methods

### Molecular dynamics simulations

Molecular dynamics simulations were run on the apo PXR-RXR LBD heterodimer and heterotetramer. MD simulations were also performed for the nuclear receptors ERα (monomer and homodimer) and PPARγ (wild-type heterodimer with RXR and mutant P467L heterodimer with RXR). A summary of these simulations containing their oligomeric states, starting structure PDB IDs, and activity is provided in Table 1. All starting structures were obtained from the protein databank (www.rcsb.org). The PXR-RXR heterodimer and heterotetramer models as proposed in Noble et al. [12] were generated by first generating a PXR-RXR heterodimer model, followed by overlaying two copies of the heterodimer onto each protomer of the PXR homodimer structure. The PXR-RXR heterodimer model was created by superimposing the PXR LBD onto the LBD of PPARγ in the PPARγ-RXRα heterodimer crystal structure (PDBID: 1FM6). Upon creating this model, the PXR LBD was found to make nearly identical salt bridges, hydrogen bonds and hydrophobic interactions with the RXRα LBD as seen in the PPARγ-RXRα heterodimer crystal structure.

All MD simulations were carried out with a 2 fs time step using the AMBER 2003 force field [34]. Molecular graphics figures were generated in Pymol (http://pymol.sourceforge.net). All production runs employed the PMEMD module from Amber 9.0 [35]. Frames were recorded every 0.4 ps. Topology and parameter files were created using the LEaP program within AMBER [35]. The simulation system consisted of the protein surrounded by a truncated octahedron of water and sodium ions to maintain charge neutrality. An explicit solvent model was used with TIP3P water molecules filling 12.5 Å between the surface of each protein and the edge of the box [36]. Electrostatic interactions were calculated using the particle-mesh Ewald algorithm [37] with a cutoff of 10 Å applied to Lennard-Jones interactions.

The SANDER package within AMBER was used for 5000 steps of energy minimization. Equilibration included 20 ps of constant volume conditions with heating from 100 to 300 K followed by 100 ps constant temperature conditions. Constant volume heating from 200 to 300 K was applied to the system for 20 ps before beginning the production run with the NPT ensemble.

Simulations were analyzed using the PTRAJ package in Amber [35]. All-atom moving average root-mean-square deviations (RMSD) were calculated for each trajectory using the initial crystal structure as reference with an interval of 100 data points. Quasiharmonic analysis was employed for each trajectory using PTRAJ [35].

### Loop modeling

In all PXR simulations, a disordered loop (PDB ID 1ILG, residues 178–197) missing from the apo PXR LBD crystal structure was modeled using the MODELLER module of InsightII with database searching (www.accelrys.com) [38]. The N and C termini of the modeled loop segment were reconnected to the missing sections of the crystal structure to avoid the termini from unrealistic flopping during simulations. The loop was examined for its potential impact on the RMSD from starting crystal structure by analyzing the simulations of the PXR LBD with and without the loop. The loop was found to impact the overall magnitude, but not the variability of the RMSD, suggesting that these regions move more than others, but do not effect stable conformations sampled during the simulation. Therefore, we have omitted the loop from subsequent analyses. However, we chose to include this loop in our simulations because it is a more realistic biological representation of the receptor.

### Correlation analysis

The pair-wise correlation coefficient as described in Sharma et al. [18], C_ij_, was computed between α-carbons of two residues, i and j, with values ranging from −1 to +1. The more positive the value of C_ij_, the more correlated (moving in the same direction with one another) the two residues, i and j, move. Likewise, the more negative the value of C_ij_, the more anticorrelated (moving in the opposite direction to one another) the two residues, i and j, move. The single-linkage clustering method [39] was applied to identify distinct sets of residues that move correlated with each other or anticorrelated to each other. In this method, a graph is initially built where each entity corresponds to individual residues. The clustering method proceeds by first finding two entities that have the highest similarity (i.e., the correlation coefficient) between them. After clustering those two entities into one, the similarities between this new entity and the rest are updated. This process is repeated until there are no more entities to cluster or the correlation coefficient cutoff is satisfied. In a single-linkage clustering method, the similarity between two clusters is defined as the largest similarity or the highest correlation coefficient between any two members from the two clusters.

### Angle analysis

Residues chosen to describe motion in Tables 2–4 were not chosen at random in the AF-2 domain of PXR LBD. Glu-427 of αAF and Lys-259 of α3 are the “charge clamp” residues of PXR; the charge clamp is a common structural motif in nuclear receptor-coactivator interactions and involves contacts between the LBD and the termini of the coactivator LxxLL helix. Lys-277 of α4 was chosen because it is conserved in many receptors and Leu-424 of αAF directly contacts the coactivator SRC-1 [14]. Angle analysis was performed using Equation 1 to find the average angle between vectors for α-carbon a and b.
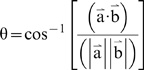
(1)


### Quasiharmonic analysis

The effective modes of vibrational motion can be obtained using quasiharmonic analysis by calculating a force field relative to the average structure based on the fluctuations generated from an MD simulation. Quasiharmonic modes, unlike standard principal component methods, are mass weighted just as normal modes and thus may be compared directly with normal mode analysis. However in quasiharmonic modes, anharmonic effects are implicitly included and thus may be different from normal modes [40]. The percent contribution of each quasiharmonic analysis mode to the overall motion can be evaluated by analyzing the eigenvalues of the first 50 modes. The percent contribution of each mode can be determined by taking the reciprocal of the eigenvalue of one mode and dividing by the sum of the inverse eigenvalues for all 50 modes. The eigenvalue is equivalent to the square of the frequency (cm^−1^). The percent contribution of each mode (Figure S5) drops off quickly with only the first few modes showing any significant contribution to the overall motion. Modes 1 and 2 in the PXR-RXR heterodimer and heterotetramer simulations represent proximal percent contributions, while in ERα and PPARγ-RXR simulations mode 2 contributed 50% less to overall motion than mode 1 (Figure S5). In order to sample the most relevant motions, the first two modes were analyzed for PXR-RXR simulations and only the first mode was analyzed in the ERα and PPARγ-RXR simulations. In all cases, the first mode(s) were sufficient to describe between 18–33% of the overall motion (Figure S5). To simplify analysis of the PXR-RXR simulations, the sums of the x, y and z vector components of each atom in each mode were obtained and weighted against the percent contribution.

### Normal mode analysis

Normal mode analysis (NMA) is based on a harmonic approximation of the potential energy function around a minimum energy conformation [41],[42]. ELNEMO uses a Hookean potential described by Tirion [41],[43], which assumes that the total energy potential function of the reference 3D structure (in this case the crystal structure) is at an energy minimum. In NMA, the lowest energy modes (below 30–100 cm^−1^) have the largest contribution to the amplitude of atomic displacements. However the first six normal or vibrational modes represent rotational and translational motion and are disregarded [44].

Normal mode theory has been shown to accurately describe large conformational transitions in proteins such as hexokinase [45], lysozyme [46],[47] and citrate synthase [48] which occur at microsecond or millisecond time scales. Fifty normal modes were generated using the ELNEMO server for each state of the three nuclear receptors [44]. The only change made was the removal of the modeled loop region (residues 178–197) in the PXR-RXR complexes, as these residues resulted in low frequency modes with low collectivity. Collectivity is a measure of the fraction of residues affected by a given mode. Computed normal modes sometimes have localized motion that corresponds to extended parts of the protein and are usually ignored [44]. This was done to confirm that the high degree of correlated motion we observed in simulations involving the active-capable forms of nuclear receptors were relevant at longer time scales.

Just as in the quasiharmonic analysis of the all-atom molecular dynamics simulations, we first sought to determine the minimum number of modes required to obtain an accurate description of the overall motion. Figure S6 shows the percent contribution of each mode, up to the first 50 modes. The first six modes of motion are trivial and have been removed from the analysis. Except for the tetramer, the percent contribution of each of the normal modes appears to drop off more slowly than those of the QH analysis (Figure S5, S6). We chose to analyze modes 7–20, which describe from 48–81% of the overall motion of each nuclear receptor (Figure S6). To simplify the analysis of the modes, we calculated the vector sum of each atom for modes 7–20, weighted by the percent contribution of each mode.

## Supporting Information

Figure S1 Conservation of Total Energy During ERα and PPARγ-RXR Simulations. Total energy (kcal/mol), used as a measure of overall simulation stability, remains relatively constant during the course of both ERα and PPARγ-RXR simulations. The final 10 ns (boxed) were used for analysis. Both the total energy (grey diamonds) and a running average (black line) are shown.
(0.48 MB TIF)

Figure S2 Root Mean Square Deviations from Starting Crystal Structures of PXR LBD Trajectories. Both the all-atom RMSD raw data (grey) and moving average (black, dashed line; blue, solid line) are plotted for PXR-RXR simulations. Both trajectories have stable RMSDs after approximately 15 ns. The most stable section of the trajectories, 20–30 ns (boxed), was used for analysis.
(2.23 MB TIF)

Figure S3 Root Mean Square Deviations from Starting Crystal Structures of ERα and PPARγ Simulations. ERα monomer, PPARγ-RXR wild-type, and PPARγ P467L-RXR simulations were stable after 10 ns; data from 10–20 ns (boxed) were used in analysis. The ERα homodimer simulation was considered stable after 15 ns; data from 15–25 ns (boxed) were used in analysis. Moving averages without raw data are plotted to provide clearer visualization.
(0.30 MB TIF)

Figure S4 Normalized Covariance Matrices for ERα and PPARγ Simulations. Correlation/anticorrelation versus secondary structure is shown for ERα monomer versus ERα homodimer (A) and the PPARγ P467L-RXR mutant heterodimer versus wild-type PPARγ -RXR heterodimer (B). Correlation coefficient values are displayed using colors ranging from blue (completely anticorrelated, −0.9) to red (completely correlated, +1) with uncorrelated residue pairs in yellow. Secondary structure is provided from left-to-right and bottom-to-top.
(12.26 MB TIF)

Figure S5 Percent Contribution to Total Motion by Each Mode of Motion Using Quasiharmonic Analysis (First 50 Modes). Modes 1 and 2 were used to analyze motion in the PXR-RXR simulations; only mode 1 was used for all other simulations.
(0.25 MB TIF)

Figure S6 Percent Contribution to Total Motion by Each Mode of Motion Using Normal Mode Analysis (First 50 modes). In normal mode analysis, the first six modes of motion are trivial and have been removed from analysis (Methods). Except for the PXR-RXR heterotetramer, the percent contribution of each of the normal modes appears to drop off more slowly than those in the quasiharmonic analysis. Modes 7–20 were used for analysis, which describe from 48–81% of the overall motion for each nuclear receptor.
(0.25 MB TIF)
