# Supplementary figures and images for: Active Nuclear Receptors Exhibit Highly Correlated AF-2 Domain Motions
Source: PLoS Comput Biol. 2008 Jul 11;4(7):e1000111. doi: 10.1371/journal.pcbi.1000111 (PMC2432469; doi:10.1371/journal.pcbi.1000111)

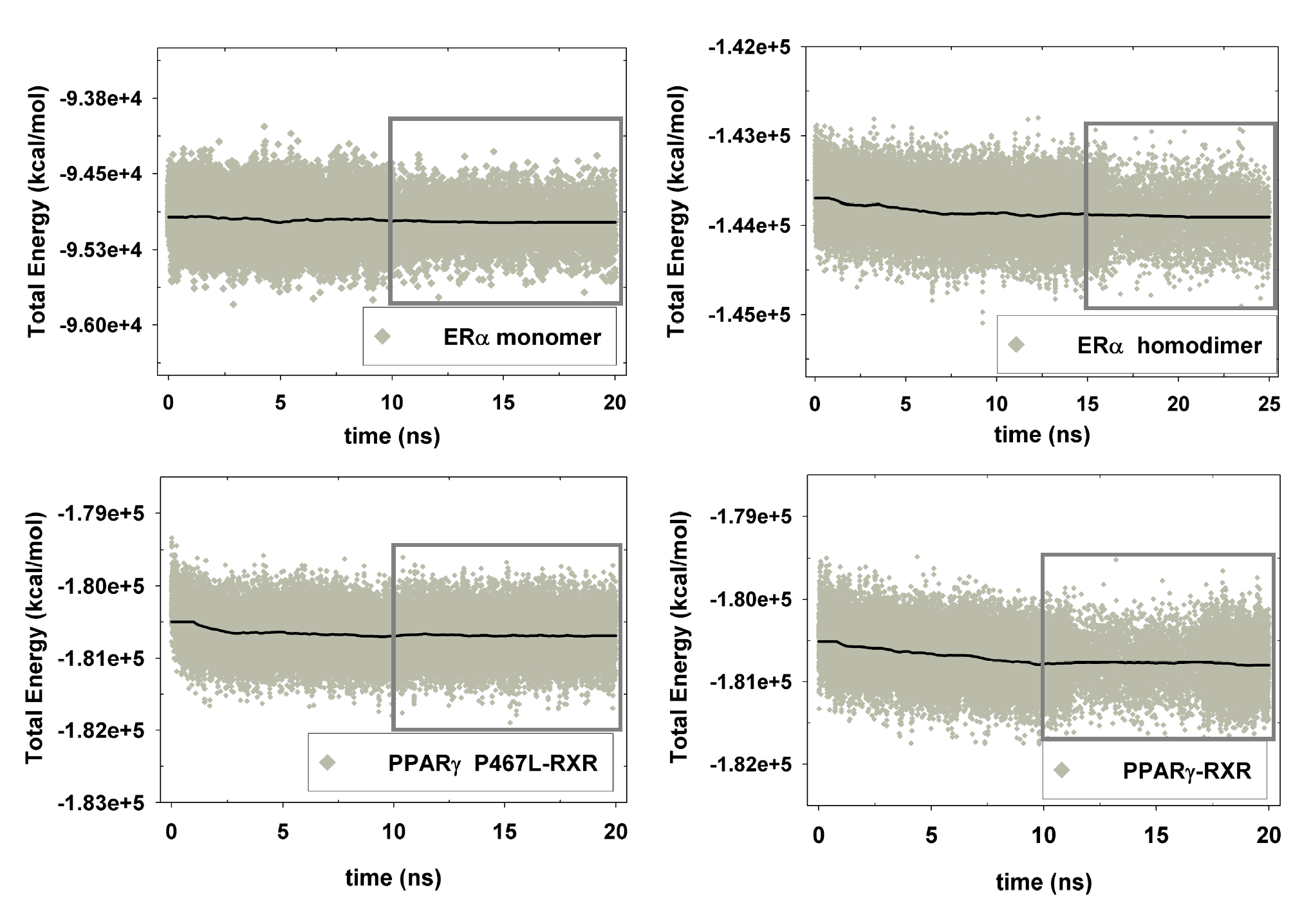

Supplement: Figure S1 — Conservation of Total Energy During ERα and PPARγ-RXR Simulations. Total energy (kcal/mol), used as a measure of overall simulation stability, remains relatively constant during the course of both ERα and PPARγ-RXR simulations. The final 10 ns (boxed) were used for analysis. Both the total energy (grey diamonds) and a running average (black line) are shown. (0.48 MB TIF) [file pcbi.1000111.s001.tif]

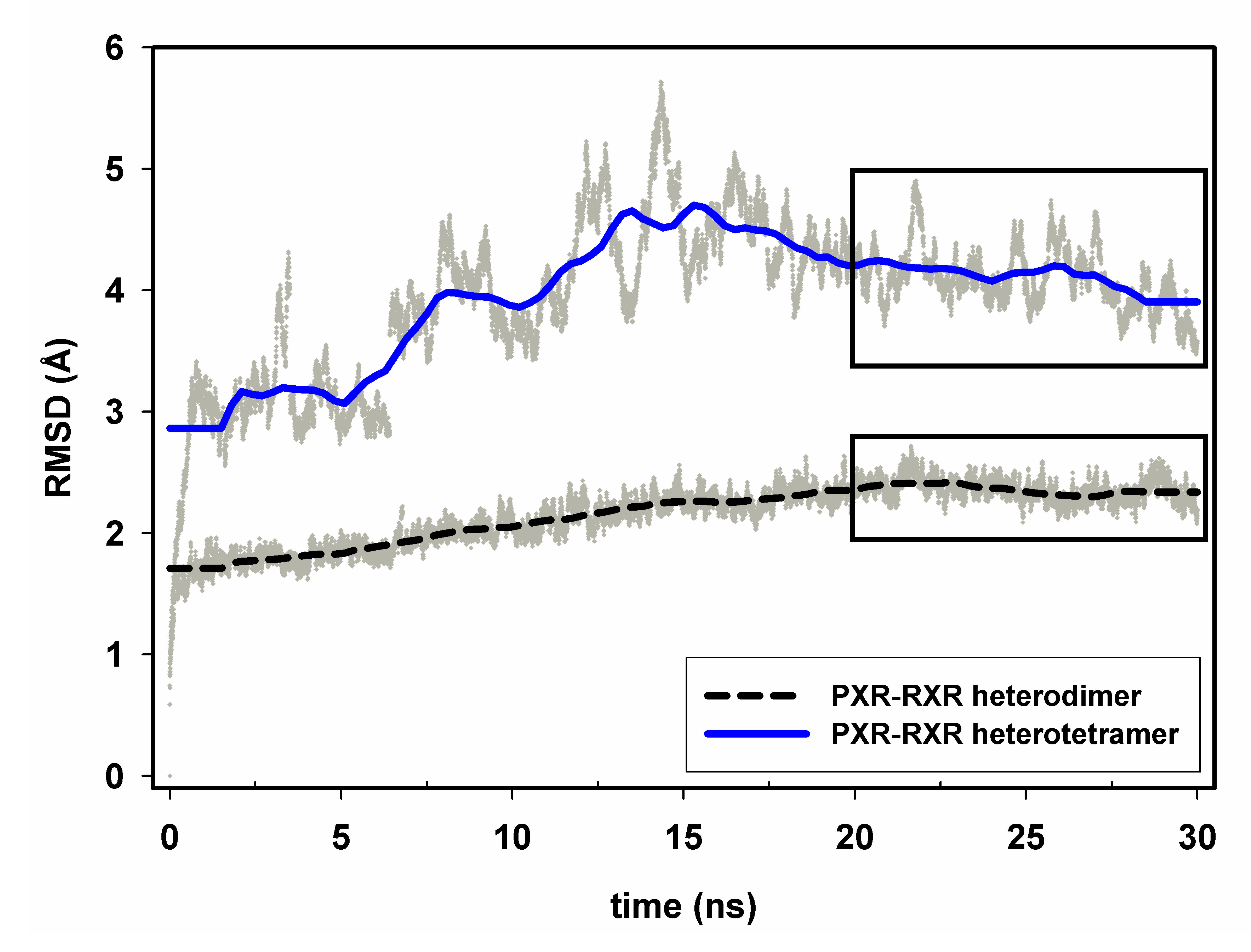

Supplement: Figure S2 — Root Mean Square Deviations from Starting Crystal Structures of PXR LBD Trajectories. Both the all-atom RMSD raw data (grey) and moving average (black, dashed line; blue, solid line) are plotted for PXR-RXR simulations. Both trajectories have stable RMSDs after approximately 15 ns. The most stable section of the trajectories, 20–30 ns (boxed), was used for analysis. (2.23 MB TIF) [file pcbi.1000111.s002.tif]

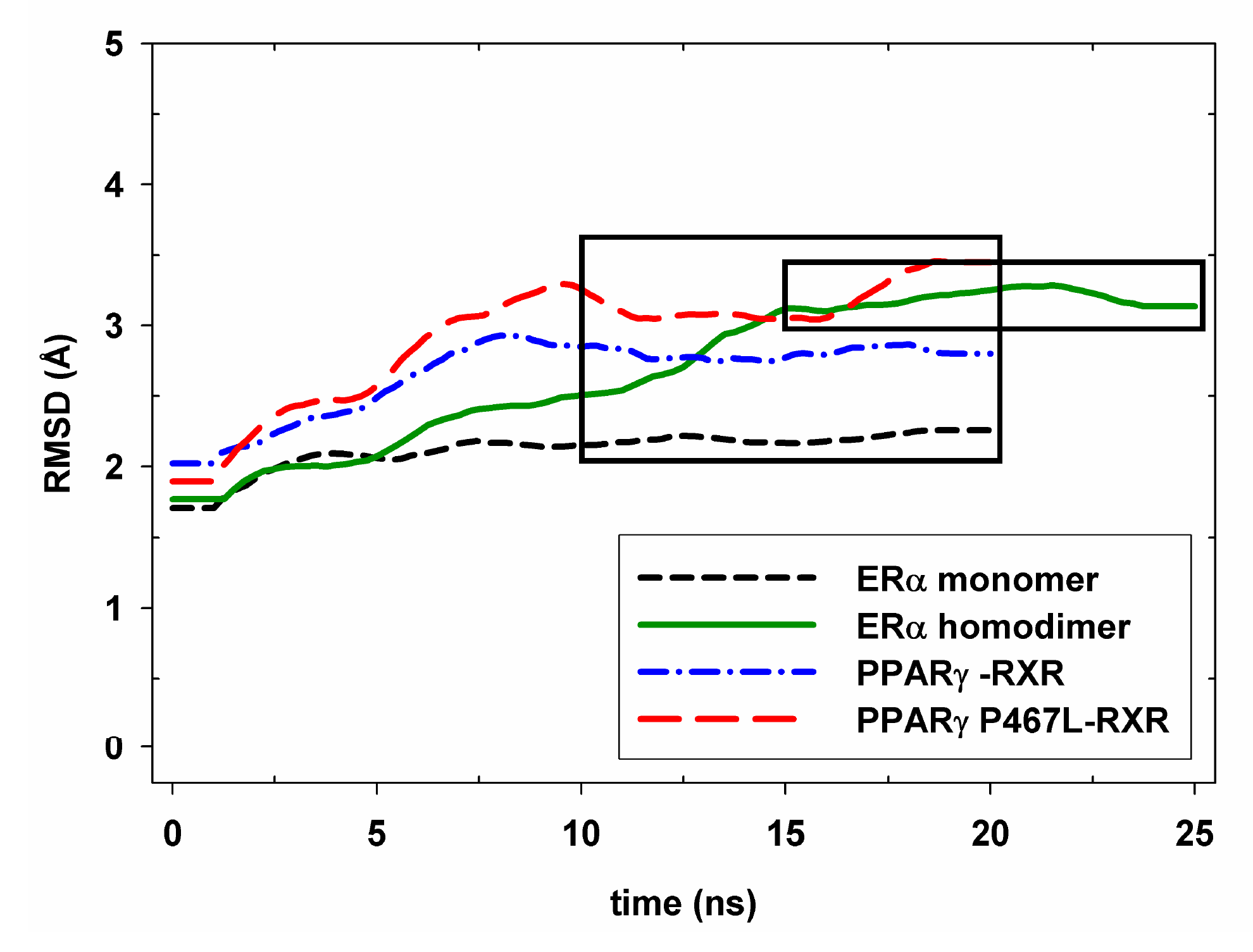

Supplement: Figure S3 — Root Mean Square Deviations from Starting Crystal Structures of ERα and PPARγ Simulations. ERα monomer, PPARγ-RXR wild-type, and PPARγ P467L-RXR simulations were stable after 10 ns; data from 10–20 ns (boxed) were used in analysis. The ERα homodimer simulation was considered stable after 15 ns; data from 15–25 ns (boxed) were used in analysis. Moving averages without raw data are plotted to provide clearer visualization. (0.30 MB TIF) [file pcbi.1000111.s003.tif]

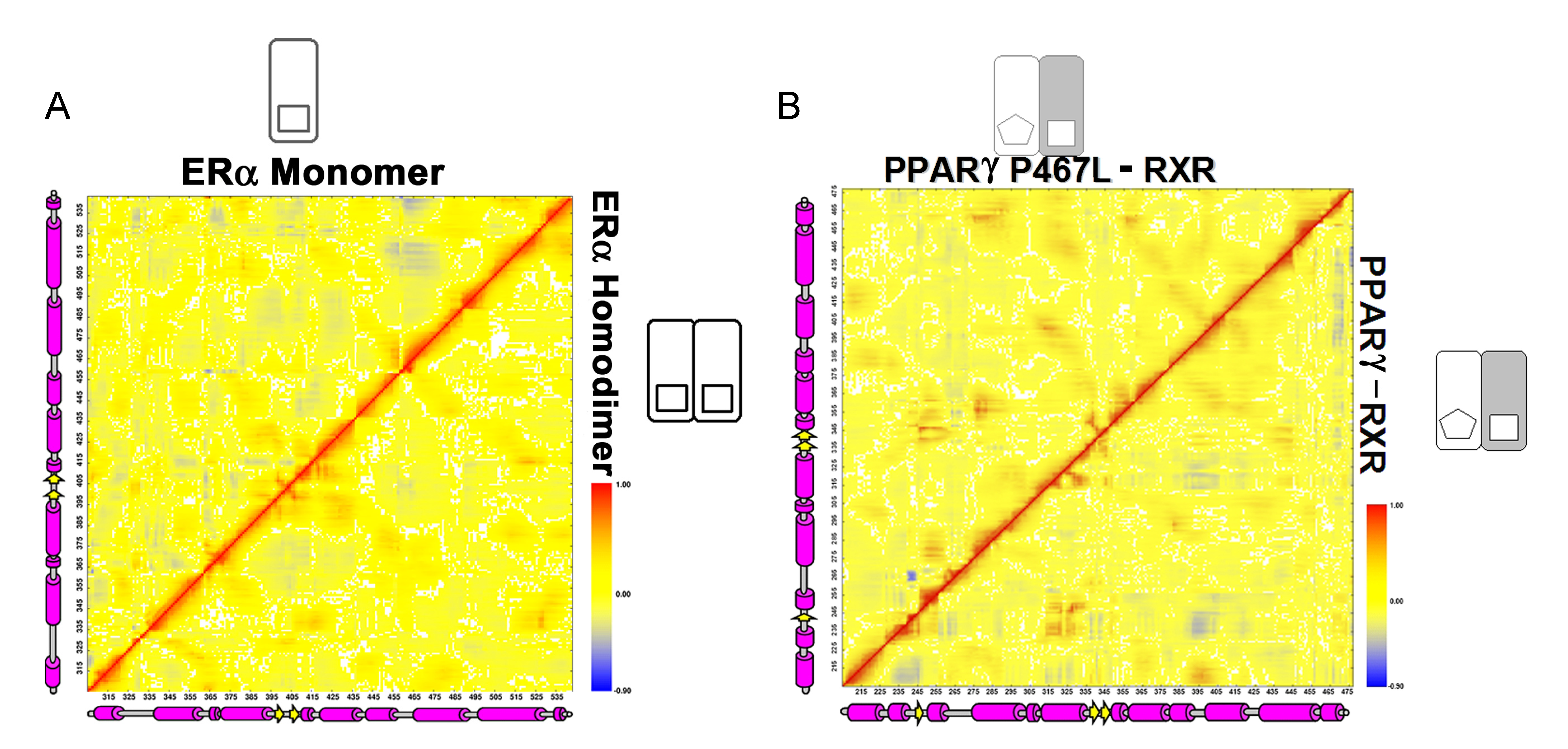

Supplement: Figure S4 — Normalized Covariance Matrices for ERα and PPARγ Simulations. Correlation/anticorrelation versus secondary structure is shown for ERα monomer versus ERα homodimer (A) and the PPARγ P467L-RXR mutant heterodimer versus wild-type PPARγ -RXR heterodimer (B). Correlation coefficient values are displayed using colors ranging from blue (completely anticorrelated, −0.9) to red (completely correlated, +1) with uncorrelated residue pairs in yellow. Secondary structure is provided from left-to-right and bottom-to-top. (12.26 MB TIF) [file pcbi.1000111.s004.tif]
